# Positive Selection of a Pre-Expansion CAG Repeat of the Human *SCA2* Gene

**DOI:** 10.1371/journal.pgen.0010041

**Published:** 2005-09-30

**Authors:** Fuli Yu, Pardis C Sabeti, Paul Hardenbol, Qing Fu, Ben Fry, Xiuhua Lu, Sy Ghose, Richard Vega, Ag Perez, Shiran Pasternak, Suzanne M Leal, Thomas D Willis, David L Nelson, John Belmont, Richard A Gibbs

**Affiliations:** 1 Human Genome Sequencing Center, Baylor College of Medicine, Houston, Texas, United States of America; 2 Broad Institute of the Massachusetts Institute of Technology, and Harvard University, Cambridge, Massachusetts, United States of America; 3 ParAllele Bioscience, South San Francisco, California, United States of America; University of Michigan, United States of America

## Abstract

A region of approximately one megabase of human Chromosome 12 shows extensive linkage disequilibrium in Utah residents with ancestry from northern and western Europe. This strikingly large linkage disequilibrium block was analyzed with statistical and experimental methods to determine whether natural selection could be implicated in shaping the current genome structure. Extended Haplotype Homozygosity and Relative Extended Haplotype Homozygosity analyses on this region mapped a core region of the strongest conserved haplotype to the exon 1 of the Spinocerebellar ataxia type 2 gene (*SCA2*). Direct DNA sequencing of this region of the *SCA2* gene revealed a significant association between a pre-expanded allele [(CAG)_8_CAA(CAG)_4_CAA(CAG)_8_] of CAG repeats within exon 1 and the selected haplotype of the *SCA2* gene. A significantly negative Tajima's D value (−2.20, *p* < 0.01) on this site consistently suggested selection on the CAG repeat. This region was also investigated in the three other populations, none of which showed signs of selection. These results suggest that a recent positive selection of the pre-expansion *SCA2* CAG repeat has occurred in Utah residents with European ancestry.

## Synopsis

Natural selection ultimately acts on the genetic variants existing among human populations. Therefore, there are “footprints” that the selective force has left behind in the human genome. In this study, Yu et al. identified an extremely large region on Chromosome 12 that is under positive selection in Utah residents with European ancestry by characterizing the correlation patterns of genomic variants. Further analyses on this interval suggested that selection centered on one of the many forms of Spinocerebellar ataxia type-2 (*SCA2*) gene. The selected form was next demonstrated to associate with one short version of the disease-causing CAG repeat in the *SCA2* gene. These results suggest that the CAG repeat was positively selected. An abnormally long version of CAGs can cause SCA2, a neurodegenerative disease that severely impairs the abilities of body movement. The authors showed how they unraveled natural selection acting on the *SCA2* gene. Their findings might lead to the discovery of the biological functions of this gene and its CAG repeat. This kind of study holds potential to facilitate the finding of common disease genes.

## Introduction

The International Haplotype Mapping (HapMap) Project [1,2] has generated a large set of evenly spaced human genomic variation data on samples from four different populations [Utah residents with ancestry from northern and western Europe (CEU); Han Chinese in Beijing, China (CHB); Japanese in Tokyo, Japan (JPT); and Yoruba in Ibadan, Nigera (YRI)]. The marker distribution on the chromosomal scale enables the identification of regions that represent locus-specific statistical deviations from overall genomic patterns. When effects from technical or sampling factors are properly considered, then regions of biological interest are revealed. With a genome-wide dense marker set, effects from ascertainment bias in marker selection are minimized, and other driving forces (e.g. drift, population expansion, migration, and non-random mating) are controlled, because they act upon all loci across the entire genome in a similar and predictable fashion [3–5]. In this study, we have demonstrated the effects of selection on a region surrounding a single allele. First, we noted a region of extensive linkage disequilibrium (LD) containing multiple single nucleotide polymorphisms (SNPs) suggesting a candidate region for natural selection on Chromosome 12 in CEU. We next applied more rigorous Extended Haplotype Homozygosity (EHH) analyses [6] to measure allelic-specific LD in this region of interest and to identify the selected genes and alleles. In order to control for recombination rate variation across the genome, the Relative EHH (REHH) test was applied, in which the different alleles in the same region serve as internal controls to normalize recombination rate variation.

A principal allele was identified within one particular haplotype spanning exon 1 of the spinocerebellar ataxia type 2 (*SCA2*) gene. The trinucleotide repeat expansion in this exon causes spinocerebellar ataxia [7], a neurodegenerative disorder typically with severe olivo-ponto-cerebellar atrophy [8,9], which affects the normal sensory/motor controlling functions. Previous studies of the triplet allele frequency distribution had shown that *SCA2* was unusual, in that the pre-expansion allele accounted for more than 90% of sampled chromosomes [10], whereas much higher triplet polymorphism rates are commonly seen in other genes that underlie neurodegenerative diseases [11]. Our results provide evidence that positive selection has favored this pre-expansion CAG repeat in the human *SCA2* gene, and that it is responsible for its overall predominance in CEU in pre-disease versions of the gene.

## Results

### Identification of One Positively Selected Region on Human Chromosome 12

We carried out large scale genotyping on human Chromosome 12 using Molecular Inversion Probe technology [12,13]. Across the four populations, on average 47,452 SNP markers were successfully genotyped and deposited in the HapMap database with a completeness, repeatability, and trio accuracy of 98.9%, 99.4%, and 99.5%, respectively. Among these SNPs, ~70% of markers have a minor allele frequency (MAF) greater than 0.05 in at least one population. The marker density on average is one SNP per ~2.8 kb, which is sufficient to enable the understanding of detailed haplotype structure of human genome.

**Figure 1:**
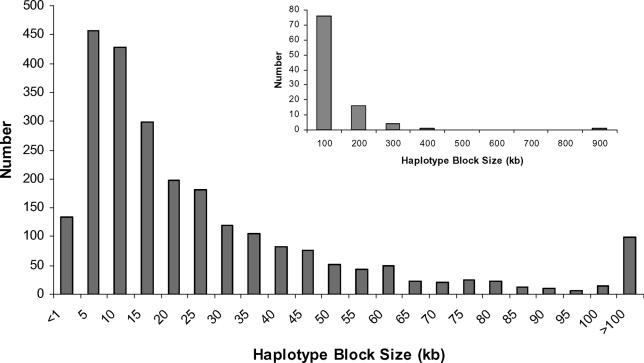
Haplotype Block Size Distribution of Human Chromosome 12 in CEU The haplotype blocks were defined by D′ confidence interval [16] and binned according to their sizes. The inset provides the ungrouped distribution of the haplotype blocks larger than 100 kb.

**Figure 2:**
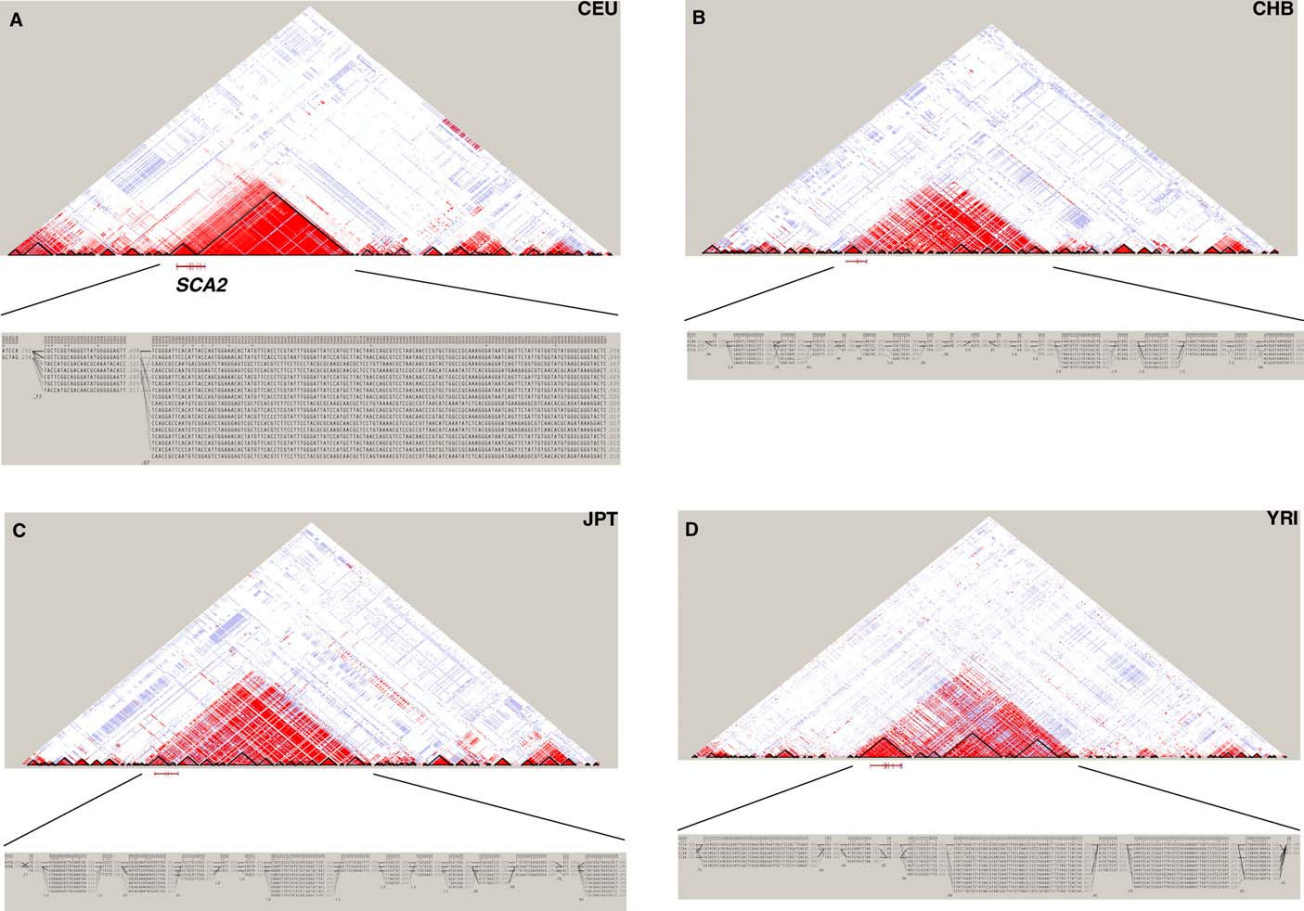
LD Patterns and Haplotypes of the Largest Block on Human Chromosome 12 in CEU (A), CHB (B), JPT (C), and YRI (D) Pair-wise LD of common SNPs (MAF > 0.05) is expressed as D′, with red indicating strong LD. The figure also shows the haplotype blocks of 1 Mb intervals in both upstream and downstream of the largest block. Schematic position of the *SCA2* gene is shown.

One effect of a recent selective sweep is a significantly large interval with strong LD around the selected site [5,14,15]. We used this effect as one criterion to detect selection across the entire Chromosome 12. In order to identify the large region with increased LD, we first constructed haplotype blocks using the LD-based empirical block definition proposed by Gabriel et al. [16]. The haplotype block size distribution of Chromosome 12 in CEU has a mean size of 26 kb, standard deviation of 43 kb, and median size of 13 kb (Figure 1). The largest block spans 987 kb however, and has 138 markers with MAF > 0.05. This is a striking outlier in the block size distribution. All studied markers in this block are in very strong LD with each other, as well as with markers in two adjacent centromeric blocks (Figure 2A), with averages of pair-wise |D′| and r^2^ of 0.91 and 0.51, respectively, that together extend the size of the overall region (110,230,654–111,393,524) in high LD to ~1.2 Mb. There are 168 SNP markers with MAF > 0.05 in this interval. The common haplotypes (> 1%, more than 2 chromosomes observed) that were inferred using Haploview [17] in the large block account for 75% of all the haplotypes, with the most common present at 30% (Figure 2A).

The global LD patterns of this region in three additional HapMap data sets (CHB, JPT, and YRI) were found to be similar, but at fine-scale, different to CEU (Figures 2B, 2C, and 2D). Specifically, there are more blocks that are shorter in length, and the LD measured by |D′| and r^2^ is weaker in the non-CEU samples (Table 1), reflecting the expected existence of more haplotype block breakpoints and historical recombination events in CHB, JPT, and YRI in this interval. This implies that the underlying haplotype structures or the evolutionary forces in this region are different in CEU when compared with the other three populations.

**Table 1:**
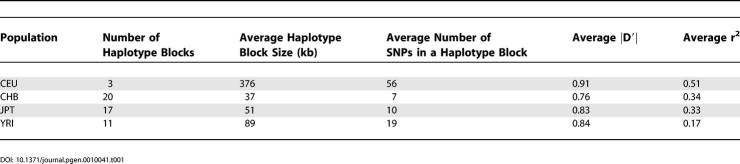
Fine-Scale Haplotype Structures and LD of the ~1.2 Mb Region in Four Populations

A possible explanation for this strong LD pattern was the presence of a large, frequent chromosomal inversion specific to CEU, as a similar structure involving a 900 kb inverted haplotype at 17q21.31 has been found in 20% of Europeans [18]. We eliminated this possibility on Chromosome 12 using PCR with primers that spanned the LD block boundary regions (111,393,230–111,397,947 and 111,428,321–111,437,985) with the possible breakpoints in 30 CEU trios (father-mother-child combinations, Table S1). We were able to predict the regions with the potential breakpoints because of the sharpness of the LD block boundaries. Because both the entire ~4 kb and the ~10 kb regions were investigated with tiled primer pairs that were designed to amplify the reference genomic sequences, at least one PCR reaction would fail to amplify any fragment in a large number of the CEU samples if an inversion breakpoint existed within this region,. In contrast, the expected fragment would be amplified in the other CEU samples without the hypothetical inversion. The amplification results revealed the expected normal fragments but no inversion breakpoints (unpublished data).

### Application of EHH and Relative EHH Analyses to Map the Causative Locus

We next sought to identify which of the multiple genes within this 1.2 Mb region (Table S2), and their positively selected alleles, might be responsible for this large region of high LD. We applied the EHH approach proposed by Sabeti et al. [6] to compare the rates of allelic specific LD decay. The EHH test exploits the assumption that under neutral evolution theory, LD of common alleles tends to be less extensive than for rare ones, due to the increased frequency of recombination and mutation as a function of the time needed for these alleles to become enriched in the population. In contrast, regions under positive selection have frequent alleles, existing on long range LD backgrounds. We also applied the REHH test, which corrects for local variation in recombination rate by comparing the EHHs of different core haplotypes present at a locus.

**Figure 3:**
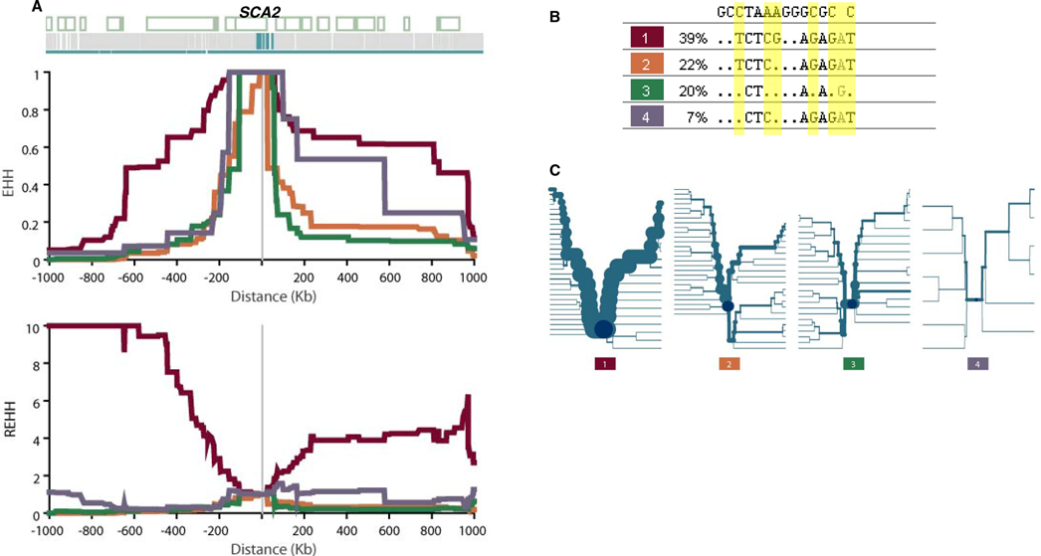
Mapping the Locus that is Under Positive Selection to the *SCA2* Gene Using the EHH Approach The mapped core region consists of 7 markers (rs593226, rs616513, rs653178, rs695871, rs3809274, rs1544396, and rs9300319). (A) The relative gene positions, EHH × distance and REHH × distance plots. (B) Four core haplotypes [TCGGGAT (39%), TCAGGAT (22%), CAACCGC (20%), and CCAGGAT (7%)]. The common SNPs are highlighted in yellow. The ancestral alleles predicted as the chimp alleles were shown on the top. (C) Haplotype bifurcation plots of the four core haplotypes.

**Figure 4:**
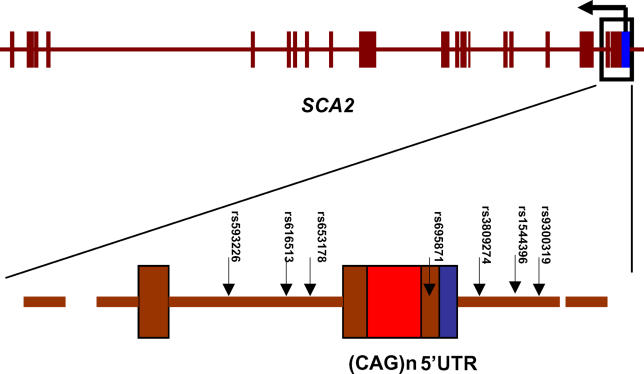
Schematic of the *SCA2* gene, (CAG)_n_, and the 7 SNPs (rs593226, rs616513, rs653178, rs695871, rs3809274, rs1544396, and rs9300319) in the Core Region

We used a sliding window approach to scan the entire 1.2 Mb interval and identify a “causative core region” (Materials and Methods). This was defined as the common core haplotype (frequency > 0.3), for which the “haplotype-specific homozogosity” was elevated when tested against distant markers, and after normalization with the recombination rate variation. Specifically, the cutoff used is that the REHH values must be greater than 2 with long-range markers, radiating to distances greater than 200 kb from the core site. This cutoff has previously been demonstrated to establish a 95^th^ percentile of significance among 5,000 simulated data sets [6]. This test identified a core region spanning seven common SNPs (Figures 3A and 3B). Among the seven SNPs, three (rs3809274, rs1544396, and rs9300319) are in the 5′ upstream of the *SCA2* gene, one (rs695871) in the 5′ coding region, while the other three (rs593226, rs616513, and rs653178) are within intron 1 (Figure 4). These data therefore led to the hypothesis that evolutionary pressure acting on the *SCA2* gene is primarily responsible for the extensive LD in the 1.2 Mb region.

**Figure 5:**
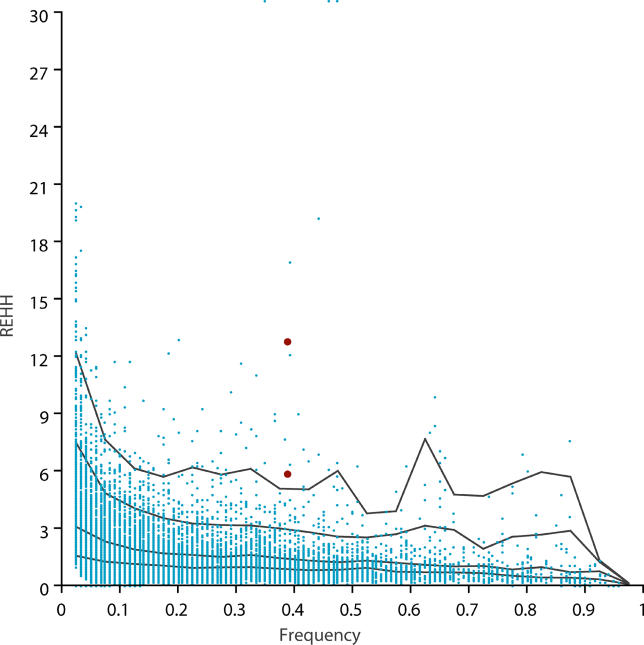
REHH × Frequency Distribution of CEU Chromosome 12 REHH was calculated at 0.25 cM distances on both sides for all possible core haplotypes from CEU Chromosome 12 and represented with blue dots and given with 50^th^, 75^th^, 90^th^, and 95^th^ percentiles. REHH values of CH-1 were represented with brown dots.

The REHH test identified one core haplotype (CH-1: TCGGGAT, frequency 39%) with elevated values over long distances (Figure 3A). CH-1 had clearly shown strikingly slower EHH decay, when compared to other haplotypes, even at a very long range. We expanded the interval by 1 Mb both upstream and downstream in an effort to detect the extended boundaries. The high EHH diminished abruptly at ~1 Mb and ~600 kb distances from the core on the proximal and distal ends, respectively (Figure 3A). At further distances from the core region, the difference of EHH values between different core haplotypes was not significant. We next used the bifurcation patterns illustrated in the diagrams to view the preserved long range homozogosity specific to each core haplotype. Each diagram is for one core haplotype, with the black dot representing the core region and each node representing one marker. The thickness of the branched lines reflects the number of samples carrying a specific haplotype. The visualized bifurcation diagram illustrates an extended predominance of one marker lineage for CH-1 (Figure 3C). To assess the significance of CH-1′s REHH values, we calculated REHH at ~0.25 centiMorgans (cM) distance on both sides from a core, for all the possible cores on Chromosome 12. We chose 0.25 cM distances for testing because it has been argued that 0.25 cM has sufficient power to detect recent (~10,000 years) selection marks [6]. The REHH values of CH-1 exceeded the 95^th^ percentile when REHH was plotted against allele frequency (Figure 5). The *p*-values for REHH calculated at CH-1′s telomeric and centromeric boundaries are 0.003 and 0.0002, respectively, when estimated by comparing with the entire Chromosome 12 distribution. Similar results were obtained when evaluated against 1,000 simulated loci [*p* = 0.0009 and *p* = 0.001 at 1 Mb telomeric and 400 kb centromeric distances from the core region. (Figure S2)]. Moreover, this core region has been recently identified as a highly significant outlier in the whole-genome REHH distribution (P. C. Sabeti, unpublished data).

The REHH analyses centered on this core region were also applied to CHB, JPT, and YRI. None of the frequent core haplotypes showed high enough REHH to satisfy our criteria (Figure S1). The results suggested that the selection associated with CH-1 is specific to CEU. Furthermore, the observation that the allele frequencies of the common SNPs in this region in CEU are quite different from the other three populations (unpublished data) also supported the existence of CEU-specific selection pressure on this region.

### Positive Selection of a Pre-Expansion CAG Repeat of the *SCA2* Gene

The reason that the *SCA2* gene would be under strong positive selection in CEU is not obvious. An expanded trinucleotide repeat expansion that codes for polyglutamine in exon 1 has been characterized as the causative mutation of progressive cerebellar ataxia. This is the most common autosomal dominant cerebellar ataxia in diverse ethnic and geographical populations [19]. The normal alleles of the CAG repeat vary in length from 14–31 triplets, and frequently include one or more CAA interruptions, whereas the disease alleles have more than 31 CAG triplets without CAA interruption. The severity of the disease and the age of onset are negatively correlated with the length of CAG repeat. Because exon 1 is within the “core” of positive selection for the 1.2 Mb region, and because it contains the causative mutation site for this locus, we hypothesized that a specific allele of the CAG trinucleotide repeat was associated with the core haplotype and potentially played an important role in selection.

In order to test this hypothesis, we both genotyped and sequenced the CAG repeat in samples from CEU, CHB, JPT, and YRI. There are 15 different *SCA2* CAG repeat alleles found in these samples (Table 2). More alleles were detected in YRI than in CEU, CHB, and JPT, and the common alleles (a-4 and a-5) found in non-Africans represent a subset of the common ones (a-4, a-5, and a-10) in YRI. Specifically, a-4 and a-5 are present at relatively high frequency in all four populations, and together account for 49%, 90%, and 100% of chromosomes in YRI, CEU, and CHB / JPT, respectively. The allele a-10 was a unique common (37%) allele found only in YRI. This observation is consistent with the known historical bottleneck for out-of-Africa populations. Another important observation is that except for two common alleles (a-4 and a-5), none of the rare alleles found in CEU are the same as the rare alleles in YRI, and almost all the rare alleles present in either CEU or YRI can be derived from the common ones with a point mutation or a slight CAG slippage. These results suggested that the rare alleles in CEU and YRI emerged after the separation of the major continental populations.

**Table 2:**
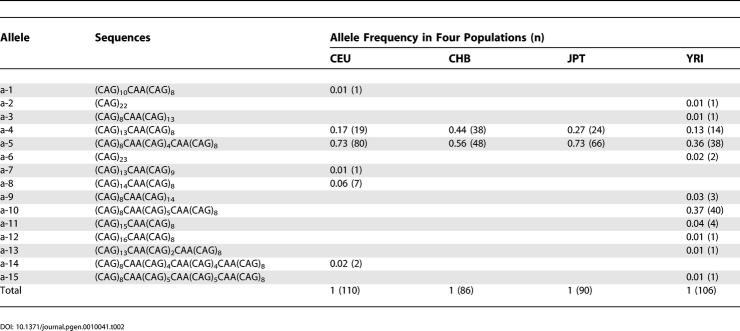
Sequencing Results of (CAG)_n_ Repeat in the *SCA2* Gene in Four Different Populations

**Figure 6:**
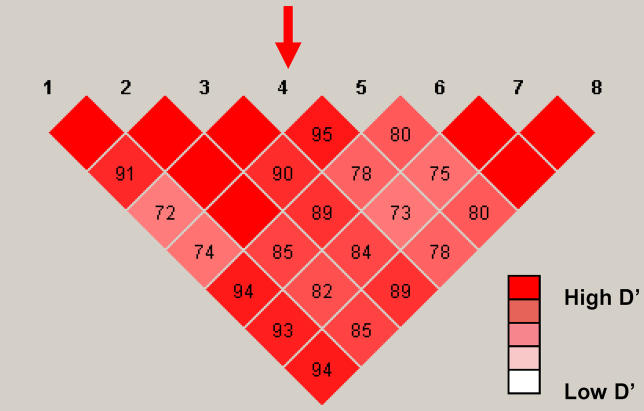
LD of CAG repeat in *SCA2* gene with the 7 Adjacent SNPs (rs593226, rs616513, rs653178, rs695871, rs3809274, rs1544396, and rs9300319) Different alleles of CAG repeat were recoded as such: 1 = (CAG)_8_CAA(CAG)_4_CAA(CAG)_8_ allele; 2 = all the other alleles; 0 = failed.

The sequenced alleles were then phased with the 7 SNPs in the core region in CEU. CH-1 is completely associated with a-5 of the CAG repeat. The a-5 allele is associated with CH-1 about 40% of the time. A chi-square test confirmed the significant correlation between CH-1 and a-5 of CAG repeat (df = 1, chi-square = 20, *p* < 0.001) in CEU. We performed a pair-wise LD test between these 7 SNP markers and the CAG repeat. The results illustrated that a-5 of the CAG repeat had a strong association with each of the other 7 SNPs (Figure 6).

The more traditional approaches, including Tajima's D-test, and Fu and Li's D*-test and F*-test, were performed to detect the selection on CAG repeat (Materials and Methods, Table S3). These test polymorphic sites and the frequency spectrum of the alleles to examine deviations from the expectations of neutral evolutionary theory. They each emphasize different characteristics: Tajima's D is sensitive to the presence of rare alleles, whereas Fu and Li's D* and F* are sensitive to singletons. These approaches are informative about the evolutionary forces in that positive selection is implied by negative values. The significantly negative Tajima's D value (*p* < 0.01) was observed for CEU, which agreed with our hypothesis that this locus has undergone a positive selection.

## Discussion

This study shows that the (CAG)_8_CAA(CAG)_4_CAA(CAG)_8_ allele (a-5) of the CAG repeat in exon 1 of the *SCA2* is significantly associated with a haplotype (CH-1) that has been detected to be under recent positive selection in CEU. As a result, a region of nearly one megabase of Chromosome 12 around this locus shows extensive LD. This is a dramatic, recent evolutionary pattern which appears to be restricted to Europeans.

Other alleles tested by the EHH approach served as internal controls to eliminate the possibilities of reduction of either the mutation or recombination rates in this physical region of chromatin or as a function of different alleles being accountable for the long LD. For example, the TCAGGAT allele is only one base different from CH-1 in the core region and only a few bases different across the entire ~1 Mb region (Figure 2A). However, its EHH decayed very quickly (Figure 3A), and showed no significance when plotted REHH values against their allele frequencies (Figure S2). These results suggest that no complication of local recombination rate variations has led to the predominance of the CH-1 haplotype. Additionally, the recombination rates (sex-average = 0.52, female = 0.87, and male = −0.11) estimated for this region using the natural logarithm of the ratio of the map distances cM × 10^6^/Mb based on deCODE and Marshfield markers do not show unusual statistical distributions, and would not account for the extremity of LD (J. Belmont et al., unpublished data).

The *SCA2* gene has an unusually low repeat variance relative to the other disease-associated coding triplet repeats [11]. The allele distribution is highly skewed towards a-4 and a-5 in CEU. Although the mutation rate has been suggested to be relatively low in this locus due to being stabilized by CAA interruptions [10], it cannot fully explain this low level of variance, because a comparable number of rare alleles were found in *SCA2* as in *SCA1* [10]. In addition, the rare alleles could easily arise from the common alleles, which implies that selective pressure could act to maintain the predominance of only a small number of alleles.

The population-specific allele spectra imply the action of other driving forces. In our study, we found that a-4 and a-5 accounted for 100% of chromosomes in CHB and JPT samples. This suggests an historical population bottleneck that could also have contributed to the formation of the 1 Mb LD observed in CEU. The chromosome-wide distribution pattern demonstrated, however, that the LD is an “outlier”, and thus it is unlikely that such factors can solely account for it. For CEU, we propose that a-4 and a-5 migrated from Africa to Europe with a-5 at much lower frequency than that found in modern Europeans, whereas the recent selective advantage on a-5 enriched it in the population. The adjacent region hitchhiked with this allele and reached high frequency quickly, therefore, the long-range LD was preserved in CEU.

The possible functional mechanisms whereby positive selection acted on the a-5 allele of the *SCA2* gene in the recent human population history are unclear. It seems unlikely that the total number of glutamine residues plays a role (a-4 and a-5 each encode 22 Gln residues), however, differences in the number of uninterrupted CAG repeats at the mRNA level could alter normal function through changes in mRNA folding and stability [20] or association with RNA binding factors. The allele a-5 shows a very low likelihood of expansion to the disease state [21], but given the late age of onset and low prevalence of the disease, it seems unlikely that disease predisposition could be directly related to the selection at this locus.

The *SCA2* gene product's normal function is unknown, although it may play a role in regulated cell death [22,23], and changes in this function could clearly be under selective constraint. Recent analysis of a *C. elegans* homologue suggests a role in translational control in the germline, another potential function under strong selective constraints [24]. Recognition of the role of selection at this locus will stimulate further investigation of the mechanism through functional studies.

It remains possible that other linked functional alterations in *SCA2* or in nearby genes on the CH-1 haplotype background were necessary for selection on a-5 or even the primary target of natural selection, with the coding triplet instead hitchhiking to high frequency.

Based on our phased results, a-5 also associates with core haplotypes other than CH-1, which do not show significantly high REHH. Other polymorphisms specific to the CH-1 allele are possibly important elements for selection. One model is that both a-5 and other unknown genetic variants on the CH-1 background each contribute modestly to the unidentified biological function, and are necessary to form a specific combination in order to confer a selective advantage. Indeed, two coding SNPs (rs695871 and rs695872) are found within 200 bp of the CAG repeat in exon 1. Our data demonstrated that a-5 is significantly associated with the rs695871 G allele, which codes for Val versus Leu. In addition, as CH-1 spans a large region (~70 kb), including the intergenic sequences between *SCA2* and *BRAP*, and the 5′-UTR and the intronic sequences of *SCA2*, the polymorphisms that might induce alternative splice sites and that might regulate differentiated expression levels of *SCA2* or the adjacent *BRAP* gene are potential candidates as well and need to be further investigated. Other genes in this ~1.2 Mb interval cannot be completely excluded as being the targets of selection even though they were not detected by the REHH analysis. For example, the *ALDH2* gene has been suspected to be selected for its hypothetical functions in resistance to endemic disease in east Asia [25]. Nevertheless, the *SCA2* is in the center of the mapped window and remains the strongest candidate gene for selection.

The uncertainty of the precise biochemical mechanism for the selection illustrates the power of the statistical genetic methods used for the identification of a “biological signal” from this locus. We expect other genomic regions to be identified in this way, and eventually to correlate the results of this kind of study with our growing understanding of biological processes.

## Materials and Methods

### Large scale SNP genotyping using Molecular Inversion Probe technology.

Our genotyping effort was carried out with Molecular Inversion Probe chemistry [12,13]. Both 2-dye and 4-dye labeling protocols for microarray based detection were used. The SNPs were allocated by the International HapMap Consortium. Assays were designed with even marker spacing and genotyped 30 trios (consisted of parents and a child) of CEU, 30 trios of YRI, 45 unrelated individuals of CHB and 45 unrelated individuals of JPT designated for the HapMap project. The data have been submitted to www.hapmap.org.

### Haplotype block definition.

NCBI build 34 and HapMap public release #16 were used as references throughout this study. The pair-wise D′ was calculated, and blocks were defined using the D′ confidence interval approach [16]. The largest block on human Chromosome 12 maps to 12q24.12 −13, and the physical coordinates are from 110,405,839–111,393.524. The detailed blocks and their underlying haplotype structure were visualized using Haploview 3.0 [17].

### CAG repeat genotyping and sequencing.

For genotyping assay, PCR amplification was performed using a pair of primers, *SCA*-A and *SCA*-B, (F, 5′- GGGCCCCTCACCATGTCG-3′; R, 5′- CGGGCTTGCGGACATTGG 3′) as previously described [7], in which *SCA*-A was 5′ end labeled with either FAM or TET. Twenty pmole each of primers were added to 25 ng of human DNA with Invitrogen's 2× multiplex mix. After an initial denaturation at 95 °C for 5 min, 36 cycles were repeated with a denaturation at 96 °C for 1.5 min, an annealing temperature of 62 °C for 30 s, an extension at 72 °C for 1.5 min, and a final extension of 5 min at 72 °C. Microsatellite genotyping was carried out by using Applied Biosystems 3730 sequencer, and the data was analyzed by using Genemapper software version 3.5 (Applied Biosystems, Foster City, California, USA). The size standard used to analyze the data was GS500 (-250LIZ). Multiple runs were analyzed for each patient and microsatellite polymorphisms were confirmed by pedigree checking.

In the sequencing assay, *SCA*-A and -B were tailed with the universal sequencing primers (Forward, 5′-CTCGTGTAAAACGACGGCCAGT-3′; Reverse, 5′-CTGCTCAGGAAACAGCTATGAC-3′). After PCR products were purified with Exo-SAP, sequencing reactions were carried out with both *SCA*-A/B and the universal primers using standard BigDye V3.1 protocol. Traces were manually analyzed using the Sequencher program.

The samples from CEU were both genotyped and sequenced. The genotyping peak lengths completely agreed with their corresponding (CAG)n sequences (unpublished data).

### Tajima's D, Fu and Li's D* and F* tests.

We sequenced 130 bases (according to the reference genomic sequence) centered on CAG repeat in *SCA2* as described above. All the polymorphisms identified were in the CAG repeats, including the CAG copy number changes that were recoded as nucleotide polymorphisms. The numbers of polymorphic sites discovered are 31, 1, 1, and 27 in CEU, CHB, JPT and YRI, respectively. We performed Tajima's D [26], and Fu and Li's D* and F* tests [27] on (CAG)_n_ using DnaSP 4.0 program [28]. The statistical significance was obtained by testing the confidence limits of the statistics (two tailed test).

### Haplotype reconstruction using HAPLORE and PHASE 2.0 programs and EHH analyses.

For the 30 CEU trios and 30 YRI trios, haplotypes were first constructed from their SNP data using the logic rules implemented in HAPLORE program [29]. PHASE 2.0 [30,31] was next used to infer the haplotypes on some markers that cannot construct unambiguous haplotypes based on the offsprings' information. For the unrelated samples from CHB and JPT, PHASE 2.0 was directly applied to infer the haplotypes.

The EHH is defined as “the probability that two randomly chosen chromosomes carrying a tested core haplotype are homozygous at all SNPs for the entire interval from the core region to the distance x.” The REHH is “the ratio of the EHH on the tested core haplotype compared with the EHH of the grouped set of core haplotypes at the region not including the core haplotype tested.”

By definition of EHH analysis, the core region needs to have almost no recombination event. Since most of the nearby markers in the 1.2 Mb interval are in strong LD with high |D′| in CEU, it allowed us to use a 4-marker sliding window as our core region with 2-marker overlap between adjacent windows to scan the entire region. The “Sweep” program (P. C. Sabeti et al., in preparation) was used for detailed EHH and REHH analysis on the identified core region.

### REHH significance estimation.

We tested the significance of REHH using two comparison datasets, empirical data from Chromosome 12 of the HapMap project Release 16 and simulations. For simulations, we generated 1000 loci of 1 MB length, calibrated to provide data consistent with a variety of measures of empirical data (i.e., F_ST_, heterozygosity, minor allele frequency distribution), and using a set of model parameters (i.e., demography, recombination rate) in accordance with current estimates (S. Schaffner et al., unpublished data).

For both comparison data sets, we placed haplotypes into 20 bins based on their frequency. We compared the REHH for each common haplotype at *SCA2* to all equally frequent haplotypes from the simulations. We obtained *p*-values by log-transforming the REHH in the bin to achieve normality, and calculating the mean and standard deviation. We carried out analysis using the Sweep software program (P. C. Sabeti et al., unpublished data).

## Supporting Information

Figure S1 REHH × Distance Plots in the Other Three Populations (A) CHB.
(B) JPT.
(C) YRI.
(34 KB PDF)

Figure S2 REHH by Frequency Plot The REHH is plotted against the core haplotype frequency at ~1 Mb telomeric (A) and ~400 kb centromeric (B) to the core region.
(1.2 MB PDF)

Table S1 PCR to Test Inversion (134 KB DOC)

Table S2 Gene List in the Largest Haplotype Block in Human Chromosome 12 (33 KB DOC)

Table S3 Tests of Selection on (CAG)_n_ of *SCA2*
(31 KB DOC)

### Accession Numbers

The Entrez Gene (http://www.ncbi.nlm.nih.gov/gquery/gquery.fcgi) accession numbers are *ALDH2* (217), *BRAP* (8315), and *SCA2* (6311).

## Abbreviations

CEU: Utah residents with ancestry from northern and western Europe
CHB: Han Chinese in Beijing, China
cM: centiMorgans
EHH: extended haplotype homozygosity
HapMap: The International Haplotype Mapping Project
JPT: Japanese in Tokyo, Japan
LD: linkage disequilibrium
MAF: minor allele frequency
REHH: relative extended haplotype homozygosity
SNP: single nucleotide polymorphism
*SCA2*: Spinocerebellar ataxia type 2
YRI: Yoruba in Ibadan, Nigeria
