# Supplementary figures and images for: Positive Selection of a Pre-Expansion CAG Repeat of the Human SCA2 Gene
Source: PLoS Genet. 2005 Sep 30;1(3):e41. doi: 10.1371/journal.pgen.0010041 (PMC1239938; doi:10.1371/journal.pgen.0010041)

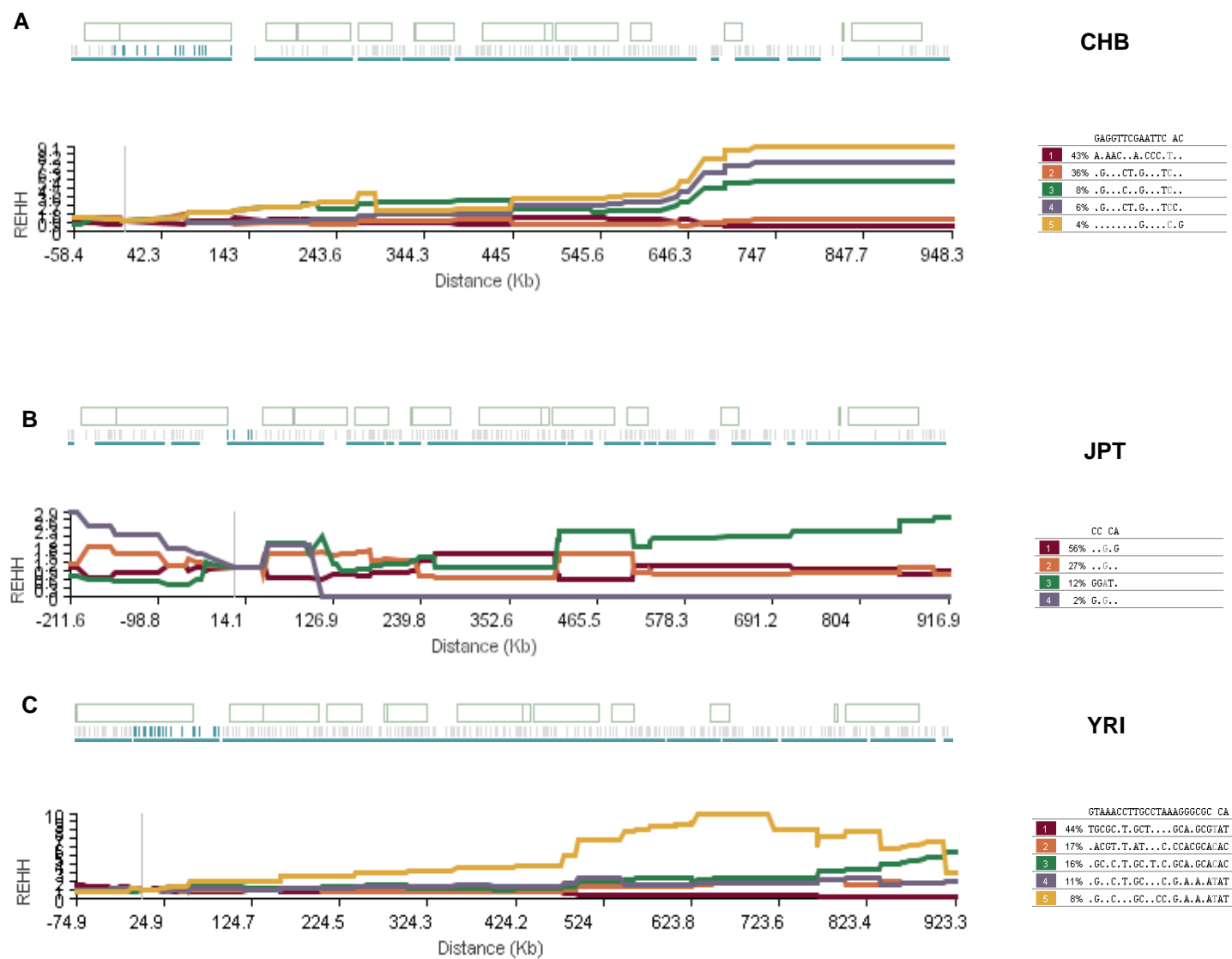

Figure S1

Supplement: Figure S1 — (A) CHB. (B) JPT. (C) YRI. (34 KB PDF) [file pgen.0010041.sg001.pdf]

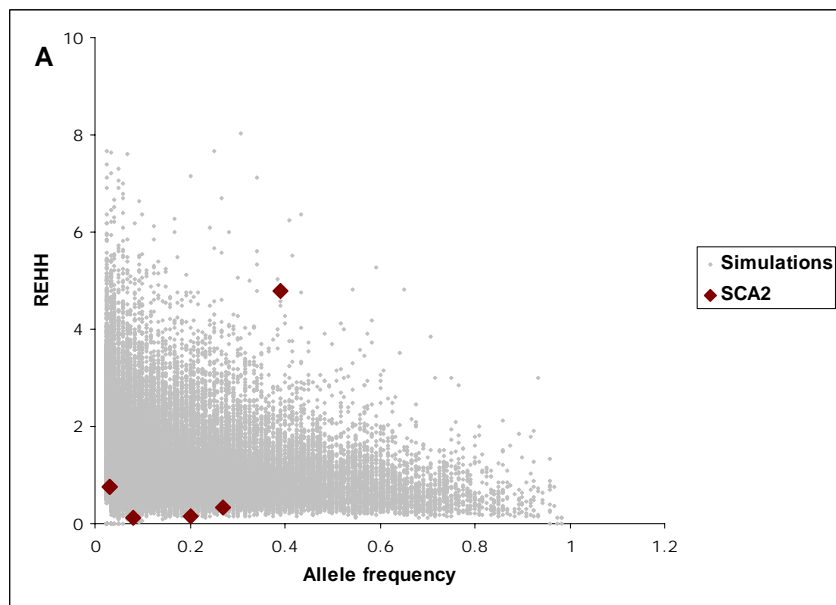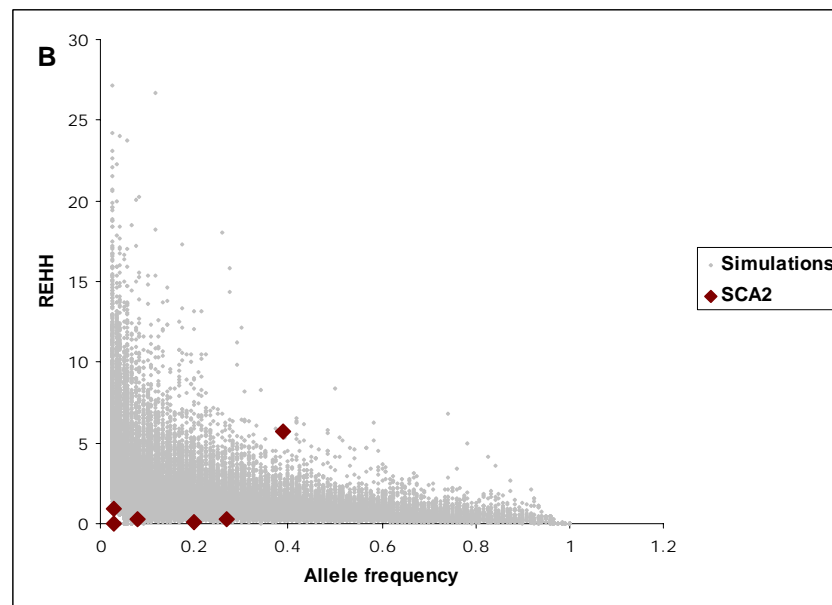

Figure S2

Supplement: Figure S2 — The REHH is plotted against the core haplotype frequency at ~1 Mb telomeric (A) and ~400 kb centromeric (B) to the core region. (1.2 MB PDF) [file pgen.0010041.sg002.pdf]
