# Supplementary material for: Positive Selection of a Pre-Expansion CAG Repeat of the Human SCA2 Gene
Source: PLoS Genet. 2005 Sep 30;1(3):e41. doi: 10.1371/journal.pgen.0010041 (PMC1239938; doi:10.1371/journal.pgen.0010041)
Supplement: Table S1 — (134 KB DOC) [file pgen.0010041.st001.doc]

| **Table S1a PCR Reactions to Test for Hypothetical Inversion in region 111393230-111397947** | | | | |
| --- | --- | --- | --- | --- |
| **Primer name** | **Sequence (5’ -3’)** | **Region** | **PCR conditions** | **Inversion** |
| FY-1AFR | TCTGCTTCCCGGATTCAA | 111393230 -  111393913 | 95°C – 5mins /( 96°C - 1.5mins/ 63°C - 0.5mins/ 72°C - 1.5mins)x36/ 72°C – 5mins/ 4°C - infinity | No |
|  | ACCCTCAAATGCATCAAACC |  |  |  |
| RV-J2FR | TTTGAGGGTGGAGACTGGAG | 111393901-  111394662 | 95°C – 5mins /( 96°C - 1.5mins/ 65°C - 0.5mins/ 72°C - 1.5mins)x36/ 72°C – 5mins/ 4°C – infinity | No |
|  | GAGTTCGAGACCAGCCTGAC |  |  |  |
| RV-J3FR | TGGACACCTCCTCCTGAGAT | 111394323-  111395095 | 95°C – 5mins /( 96°C - 1.5mins/ 63°C - 0.5mins/ 72°C - 1.5mins)x36/ 72°C – 5mins/ 4°C – infinity | No |
|  | GGCCTTCCAGGTCCTTATTC |  |  |  |
| RV-J9FR | GTCAGGCTGGTCTCGAACTC | 111394643-  111395095 | 95°C – 5mins /( 96°C - 1.5mins/ 60°C - 0.5mins/ 72°C - 1.5mins)x36/ 72°C – 5mins/ 4°C - infinity | No |
|  | GGCCTTCCAGGTCCTTATTC |  |  |  |
| RV-J10FR | TGTCATCCTACCTTGGCACA | 111395049-  111395754 | 95°C – 5mins /( 96°C - 1.5mins/ 60°C - 0.5mins/ 72°C - 1.5mins)x36/ 72°C – 5mins/ 4°C – infinity | No |
|  | CTTAGCCTCCCAAAGTGCTG |  |  |  |
| RV-J11FR | GGTGGCATGGTTCACTCTTT | 111395326-  111396052 | 95°C – 5mins /( 96°C - 1.5mins/ 60°C - 0.5mins/ 72°C - 1.5mins)x36/ 72°C – 5mins/ 4°C – infinity | No |
|  | ACCACCACCACAGAGACTCC |  |  |  |
| RV-J13FR | CAACCCCACCTGAGGTCTTA | 111395633-  111396110 | 95°C – 5mins /( 96°C - 1.5mins/ 62°C - 0.5mins/ 72°C - 1.5mins)x36/ 72°C – 5mins/ 4°C – infinity | No |
|  | GAGGACTGGCCATACTTCCA |  |  |  |
| RV-J14FR | CTGCAGCTAGGGGTCTTTTG | 111395664-  111396303 | 95°C – 5mins /( 96°C - 1.5mins/ 63°C - 0.5mins/ 72°C - 1.5mins)x36/ 72°C – 5mins/ 4°C – infinity | No |
|  | TCCACTGTTGTTCCCATTGA |  |  |  |
| RV-J15FR | GGAGTCTCTGTGGTGGTGGT | 111396033-  111396624 | 95°C – 5mins /( 96°C - 1.5mins/ 63°C - 0.5mins/ 72°C - 1.5mins)x36/ 72°C – 5mins/ 4°C – infinity | No |
|  | CACAGTGGCTCATGCCTCTA |  |  |  |
| RV-J6FR | GGAACAACAGTGGAGGGAAA | 111396290-  111397045 | 95°C – 5mins /( 96°C - 1.5mins/ 63°C - 0.5mins/ 72°C - 1.5mins)x36/ 72°C – 5mins/ 4°C – infinity | No |
|  | CATGAATGGGCTCTCAGGAT |  |  |  |
| RV-J7FR | GGATAGGGATGGGAGTGGTT | 111396822-  111397600 | 95°C – 5mins /( 96°C - 1.5mins/ 63°C - 0.5mins/ 72°C - 1.5mins)x36/ 72°C – 5mins/ 4°C – infinity | No |
|  | AGCCTGGGTGACAGAACAAG |  |  |  |
| RV-J8FR | GTCTTGGGAAGCGAATGAGA | 111397190-  111397947 | 95°C – 5mins /( 96°C - 1.5mins/ 63°C - 0.5mins/ 72°C - 1.5mins)x36/ 72°C – 5mins/ 4°C - infinity | No |
|  | CTATCCTGTCCCGAGCTGAG |  |  |  |

| **Table S1b PCR Reactions to Test for Hypothetical Inversion in region 111428321-111437985** | | | | |
| --- | --- | --- | --- | --- |
| **Primer name** | **Sequence (5’ -3’)** | **Region** | **PCR conditions** | **Inversion** |
| FY-1BFR | GTTCTGCCTGAACCAAAACA | 111428321 –  111429168 | 95°C – 5mins /( 96°C - 1.5mins/ 63°C - 0.5mins/ 72°C - 1.5mins)x36/ 72°C – 5mins/ 4°C - infinity | No |
|  | ATACGGGTGCCCACAGATTA |  |  |  |
| FY-2BFR | TCTCAAGGGCAGAAGGAATG | 111428879 –  111429577 | 95°C – 5mins /( 96°C - 1.5mins/ 62°C - 0.5mins/ 72°C - 1.5mins)x36/ 72°C – 5mins/ 4°C – infinity | No |
|  | GAGGTCAAGGCTGCAGTGAG |  |  |  |
| FY-3BFR | TCCCACCATTTGGAAAACTC | 111429229 –  111430044 | 95°C – 5mins /( 96°C - 1.5mins/ 59°C - 0.5mins/ 72°C - 1.5mins)x36/ 72°C – 5mins/ 4°C – infinity | No |
|  | GGGGGTGGGGAGATATAGAA |  |  |  |
| FY-4BFR | TCTCTTTGCCTCTTTGTCTCTG | 111429914 –  111430587 | 95°C – 5mins /( 96°C - 1.5mins/ 59°C - 0.5mins/ 72°C - 1.5mins)x36/ 72°C – 5mins/ 4°C – infinity | No |
|  | TCACATCCAATCTGCACAAA |  |  |  |
| FY-5BFR | CGTGTAAGCCAGGATGGTCT | 111430364 –  111431054 | 95°C – 5mins /( 96°C - 1.5mins/ 63°C - 0.5mins/ 72°C - 1.5mins)x36/ 72°C – 5mins/ 4°C – infinity | No |
|  | CGCACATGCTCTCTCTATGG |  |  |  |
| FY-6BFR | TCCTTGGTTACTTCCTGCAA | 111430814 –  111431513 | 95°C – 5mins /( 96°C - 1.5mins/ 61°C - 0.5mins/ 72°C - 1.5mins)x36/ 72°C – 5mins/ 4°C – infinity | No |
|  | GGCATGGTATAGAGGGAAGG |  |  |  |
| FY-7BFR | GCTTGGGAGGTAGCTAGAGATG | 111431325 –  111431994 | 95°C – 5mins /( 96°C - 1.5mins/ 62°C - 0.5mins/ 72°C - 1.5mins)x36/ 72°C – 5mins/ 4°C – infinity | No |
|  | CATGAAGACAGGCAAGACACA |  |  |  |
| FY-8BFR | GGGAAAACTCATTAAACAATTTGG | 111431771 –  111432451 | 95°C – 5mins /( 96°C - 1.5mins/ 62°C - 0.5mins/ 72°C - 1.5mins)x36/ 72°C – 5mins/ 4°C - infinity | No |
|  | AGCCCAAGAGTTCAAGACCA |  |  |  |
| FY-9BFR | CACTGTAGCCTTGACCTCCTG | 111432277 –  111433056 | 95°C – 5mins /( 96°C - 1.5mins/ 58°C - 0.5mins/ 72°C - 1.5mins)x36/ 72°C – 5mins/ 4°C - infinity | No |
|  | GGGCTGTTGTAAGGATGGAA |  |  |  |
| FY-10BFR | GTGGCTGGGAGGTAACTCTG | 111432839 –  111433625 | 95°C – 5mins /( 96°C - 1.5mins/ 63°C - 0.5mins/ 72°C - 1.5mins)x36/ 72°C – 5mins/ 4°C – infinity | No |
|  | GACTACAGGCATCTGCCACA |  |  |  |
| FY-11BFR | GTGGCCTTGAGCAAACTACA | 111433395 –  111434243 | 95°C – 5mins /( 96°C - 1.5mins/ 63°C - 0.5mins/ 72°C - 1.5mins)x36/ 72°C – 5mins/ 4°C – infinity | No |
|  | ATGTGACCCTGGCTGGAC |  |  |  |
| FY-12BFR | GGGTCTCTTTGGGAATGGAT | 111433970 –  111434776 | 95°C – 5mins /( 96°C - 1.5mins/ 63°C - 0.5mins/ 72°C - 1.5mins)x36/ 72°C – 5mins/ 4°C – infinity | No |
|  | CTTTGACCGCTGACTGAAGG |  |  |  |
| FY-13BFR | CCGGAAGGAATGAACAAAAC | 111434487 –  111435270 | 95°C – 5mins /( 96°C - 1.5mins/ 63°C - 0.5mins/ 72°C - 1.5mins)x36/ 72°C – 5mins/ 4°C – infinity | No |
|  | ATCCACCTGTCTCGAACTCC |  |  |  |
| FY-14BFR | CCTTCAATCCAAGTCAGTTTCAG | 111435073 –  111435907 | 95°C – 5mins /( 96°C - 1.5mins/ 63°C - 0.5mins/ 72°C - 1.5mins)x36/ 72°C – 5mins/ 4°C – infinity | No |
|  | CTCTGAGCTCCTCACCTGCT |  |  |  |
| FY-15BFR | GCAGGTGCCTCAAGGTAAGT | 111435744 –  111436593 | 95°C – 5mins /( 96°C - 1.5mins/ 63°C - 0.5mins/ 72°C - 1.5mins)x36/ 72°C – 5mins/ 4°C – infinity | No |
|  | ACCCTGAGGTCCAAATCCTC |  |  |  |
| FY-16BFR | AGAAGACCAAGCAGGGGAAT | 111436307 –  111437151 | 95°C – 5mins /( 96°C - 1.5mins/ 63°C - 0.5mins/ 72°C - 1.5mins)x36/ 72°C – 5mins/ 4°C - infinity | No |
|  | TTCCAGAGCTCATGATTGTCA |  |  |  |
| FY-17BFR | CTTGGGTCAAAGGGAAATCA | 111436972 –  111437784 | 95°C – 5mins /( 96°C - 1.5mins/ 63°C - 0.5mins/ 72°C - 1.5mins)x36/ 72°C – 5mins/ 4°C - infinity | No |
|  | GCATGAGCAGGAAACACAGA |  |  |  |
| FY-18BFR | ACGGCACATCTGTTGAATGA | 111437550 –  111437985 | 95°C – 5mins /( 96°C - 1.5mins/ 63°C - 0.5mins/ 72°C - 1.5mins)x36/ 72°C – 5mins/ 4°C – infinity | No |
|  | CATTTCCACAGTCCACTCCA |  |  |  |
