# Supplementary material for: Positive Selection of a Pre-Expansion CAG Repeat of the Human SCA2 Gene
Source: PLoS Genet. 2005 Sep 30;1(3):e41. doi: 10.1371/journal.pgen.0010041 (PMC1239938; doi:10.1371/journal.pgen.0010041)
Supplement: Table S2 — (33 KB DOC) [file pgen.0010041.st002.doc]

| **Table S2 Gene List in the Largest Haplotype Block in Human Chromosome 12** | | |
| --- | --- | --- |
| **Gene**  **(Entrez GeneID)** | **Full Name** | **Comments** |
| *LNK* (10019) | Lymphocyte adaptor protein | Human LNK was cloned by screening a Jurkat cell cDNA library. It has an extended coding region at the 5’ end resulting in an additional 267 amino-terminal amino acids compared with the rat and mouse sequences. Lnk mRNA is preferentially expressed in lymph mode and spleen lymphocytes. The overexpression of hLnk in Jurkat cell inhibits anti-CD3-mediated activation of NF-AT transcription activity [1]. |
| *SCA2* (6311) | spinocerebellar ataxia type 2 | The spinocerebellar ataxia (SCA) are a genetically heterogeneous group of neurodegenerative disorders characterized by progressive degeneration of the cerebellum, brain stem and spinal cord. The *SCA2* locus has been determined that the diseased allele contains 36-52 CAG repeats, compared to 22-23 in the normal allele [2]. |
| *BRAP* (8315) | BRCA1-associated protein | The protein encoded by this gene was identified by its ability to bind to the nuclear localization signal of BRCA1 and other proteins. It is a cytoplasmic protein which may regulate nuclear targeting by retaining proteins with a nuclear localization signal in the cytoplasm [2]. |
| *ALDH2* (217) | mitochondrial aldehyde dehydrogenase2 | Aldehyde dehydrogenase is the second enzyme of the major oxidative pathway of alcohol metabolism. Cytosolic and mitochondrial are the two major liver isoforms of this enzyme. Most Caucasians have two major isozymes, while approximately 50% of Orientals have only the cytosolic isozyme, missing the mitochondrial isozyme. A remarkably higher frequency of acute alcohol intoxication among Orientals than among Caucasians could be related to the absence of the mitochondrial isozyme [2]. |
| *MAPKAPK5* (8550) | MAPK-activated protein kinase | The protein encoded by this gene is a member of the serine/threonine kinase family. In response to cellular stress and proinflammatory cytokines, this kinase is activated through its phosphorylation by MAP kinases including MAPK1/ERK, MAPK14/p38-alpha, and MAPK11/p38-beta. In vitro, this kinase phosphorylates heat shock protein HSP27 at its physiologically relevant sites. Two alternately spliced transcript variants of this gene encoding distinct isoforms have been reported [2]. |

References:

1. Li Y., He X., Schembri-King J., Jakes S., Hayashi J. (2000) Cloning and characterization of human Lnk, an adaptor protein with pleckstrin homology and Src homology 2 domains that can inhibit T cell activation. ***J. Immunol***. 164:5199-5206.
2. Entrez Gene: http://www.ncbi.nih.gov/entrez/query.fcgi?db=gene.
