# Supplementary material for: Positive Selection of a Pre-Expansion CAG Repeat of the Human SCA2 Gene
Source: PLoS Genet. 2005 Sep 30;1(3):e41. doi: 10.1371/journal.pgen.0010041 (PMC1239938; doi:10.1371/journal.pgen.0010041)
Supplement: Table S3 — (31 KB DOC) [file pgen.0010041.st003.doc]

| **Table S3 Tests of Selection on (CAG)_n_ of *SCA2*** | | | | | | |
| --- | --- | --- | --- | --- | --- | --- |
| **Population** | **Tajima’s D** | ***P*(D)** | **Fu and Li’s D*** | ***P*(D*)** | **Fu and Li’s F*** | ***P*(F*)** |
| **CEU** | **-2.20** | **<0.01** | **-1.05** | **>0.10** | **-1.81** | **>0.10** |
| **CHB** | **1.78** | **>0.05** | **0.50** | **>0.10** | **1.03** | **>0.10** |
| **JPT** | **1.18** | **>0.10** | **0.50** | **>0.10** | **0.82** | **>0.10** |
| **YRI** | **-1.31** | **>0.10** | **-2.10** | **>0.05** | **-2.14** | **>0.05** |
